# Lattice surgery realized on two distance-three repetition codes with superconducting qubits

**DOI:** 10.1038/s41567-025-03090-6

**Published:** 2026-01-30

**Authors:** Ilya Besedin, Michael Kerschbaum, Jonathan Knoll, Ian Hesner, Lukas Bödeker, Luis Colmenarez, Luca Hofele, Nathan Lacroix, Christoph Hellings, François Swiadek, Alexander Flasby, Mohsen Bahrami Panah, Dante Colao Zanuz, Markus Müller, Andreas Wallraff

**Affiliations:** 1 Department of Physics, ETH Zurich, Zurich, Switzerland; 2 ETH Zurich – PSI Quantum Computing Hub, Paul Scherrer Institute, Villigen, Switzerland; 3 Quantum Center, ETH Zurich, Zurich, Switzerland; 4 Institute for Theoretical Nanoelectronics (PGI-2), Forschungszentrum Jülich, Jülich, Germany; 5 Institute for Quantum Information, RWTH Aachen University, Aachen, Germany

**Keywords:** Qubits, Quantum information

## Abstract

Quantum error correction is needed for quantum computers to be capable of executing algorithms using hundreds of logical qubits in a fault-tolerant manner. Recent experiments have progressed towards this by demonstrating sufficiently low error rates for state preservation of a single logical qubit. However, quantum computation algorithms also require that these logical qubits can be entangled and that gate operations can be performed on them. Lattice surgery is a technique that offers a practical approach for implementing such gates, particularly in planar quantum processor layouts. Here we demonstrate lattice surgery between two distance-three repetition-code qubits by splitting a single distance-three surface-code qubit. Using a quantum circuit that is fault-tolerant for bit-flip errors, we achieve an improvement in the value of the decoded *ZZ* logical two-qubit observable compared with a similar non-encoded circuit. We therefore demonstrate the functional building blocks needed for lattice-surgery operations on larger-distance codes based on superconducting circuits.

## Abstract (web summary)

Quantum error correction codes protect quantum information, but running algorithms also requires the ability to perform gates on logical qubits. A lattice surgery scheme for fault-tolerant gates has now been demonstrated in a quantum repetition code.

## Main

Scalable quantum computing relies on the ability to correct errors that may occur during computation^1^. Topological error correction codes enable the encoding of logical qubits using physical data qubits arranged in a lattice^2,3^, where physical errors in the code are detected using local stabilizer measurements^4,5^. In planar codes, such as the surface code^6^, the stabilizer measurement circuit involves only gates between neighbouring qubits, making it well suited for architectures with two-dimensional local connectivity, such as superconducting qubits.

In such codes, repeated stabilizer measurements enable the detection and correction of errors, as demonstrated in logical state preservation experiments using surface codes^7–9^ and colour codes^10–12^. Provided the syndrome extraction circuit operates with a sufficiently low error rate, experiments have shown that increasing the size of the qubit lattice, and thereby the code distance, enhances the protection of the logical state^13,14^.

Beyond state preservation, an essential component of fault-tolerant quantum computing is the ability to perform operations on logical qubits. This includes single-qubit gates, a subset of which can be straightforwardly realized transversally in some codes like the colour code^15^, as well as entangling gates between logical qubits. These can also be realized transversally, as demonstrated between distinct distance-three colour codes in systems with reconfigurable connectivity, such as trapped ions^11,15^ and Rydberg atoms^13^. However, in systems with fixed local connectivity, this approach cannot be directly applied.

Lattice surgery^16^ is a powerful technique that extends topological codes to multiple logical qubits, enabling fault-tolerant gate operations while maintaining a two-dimensional arrangement of physical qubits. The fundamental operation in lattice surgery involves measuring an observable of two logical qubits, which is executed through merge and split code deformations^17^. These two operations can be used as building blocks for more complex operations such as logical CNOT gates^18,19^ or magic state distillation^20^. Lattice surgery primitives have been successfully demonstrated in state teleportation experiments utilizing distance-two surface codes^21^ and distance-three colour codes^11,12^, as well as in a logical Bell state preparation experiment involving distance-four 3CX and Bacon–Shor codes^22^.

Here, we demonstrate lattice surgery operation on a quantum device consisting of 17 qubits realized with superconducting circuits. Specifically, we focus on the split operation applied to a rotated distance-three surface code, which results in an entangled state of two independently operated logical qubits, encoded with bit-flip repetition codes. The protocol is fault-tolerant for bit-flip but not for phase-flip errors, which remain undetectable after the split. By focusing on the boundary region connecting the two logical qubits, we investigate critical aspects of error correction and lattice surgery, such as syndrome correlation analysis during code deformation and logical process tomography, providing insights that can be applied to larger, fully fault-tolerant error-correcting schemes. This work takes a crucial step towards scalable fault-tolerant quantum computing by implementing an entangling operation between logical qubits encoded in a surface-code lattice using lattice surgery. Beyond this conceptual advance, we highlight the role of logical characterization techniques as key tools for benchmarking the performance of such operations in realistic, noisy devices.

## Lattice surgery on a distance-three surface code

**Fig. 1:**
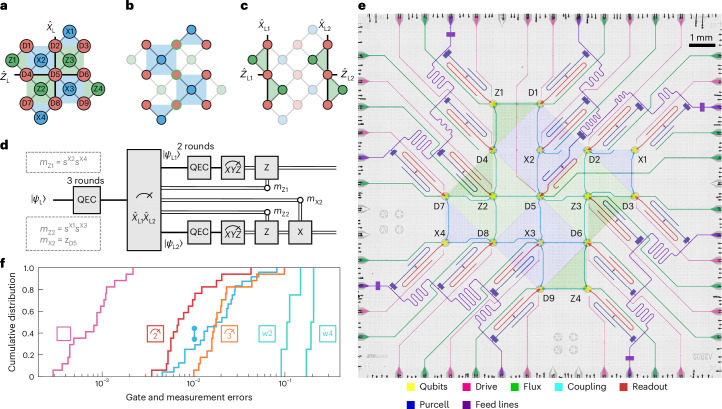
Experiment concept, quantum device layout and performance. **a**–**c**, Conceptual qubit layout and connectivity. The red circles correspond to data qubits, while the green (blue) plaquettes and circles depict *Z*-type (*X*-type) stabilizers and auxiliary qubits associated with them. The logical operator definitions of the distance-three surface code (**a**) and the two distance-three repetition codes (**c**) are indicated as solid black lines. Mid-circuit data qubit readout during the lattice-split operation (**b**) is depicted as green circle outlines. **d**, Schematic experimental sequence and Pauli-frame-update definitions *m*_Z1_, *m*_Z2_ and *m*_X2_. Double lines denote an update of the results of the tomography readouts in postprocessing based on measurement outcomes obtained during the lattice split operation. **e**, False-coloured optical photograph of the quantum chip. The stabilizer plaquettes are shown with blue and green triangles and squares. **f**, Experimental cumulative distributions of the operation errors of the device: single-qubit gates (pink), two-qubit CZ gates (cyan), readout with two-state (red) and three-state (orange) discrimination and cycle-average syndrome elements for weight-two and weight-four stabilizers (w2 and w4).

In lattice surgery^16,17^, logical qubits are encoded in patches of distinct data qubits forming a larger underlying lattice. For the surface code, this lattice is square. In each logical qubit, errors are identified through measurements of stabilizers, that is, products of Pauli-*Z* or Pauli-*X* operators acting on data qubits located at the vertices of the lattice plaquettes, with *X*-type and *Z*-type stabilizers arranged in a checkerboard pattern. The rotated distance-three surface code implemented in this work consists of a three-by-three grid of data qubits, denoted D*j*, *j* ∈ {1, …, 9}, and eight stabilizers. Each stabilizer is assigned a unique auxiliary qubit for its measurement. The qubit layout and connectivity is shown in Fig. 1a.

Error correction is performed by measuring repeated cycles of stabilizers. We denote the stabilizer operators as ${\hat{S}}^{Ai}$, and their measurement outcomes in cycle *N* as ${s}_{N}^{Ai}=\pm 1$, where *A* ∈ {*X*, *Z*} indicates the stabilizer type and *i* enumerates the stabilizers of one type. Syndrome elements are defined as the parity of stabilizer outcomes across two consecutive cycles, where a value of zero indicates even parity, and a value of one indicates odd parity: ${\sigma }_{N}^{Ai}=(1-{s}_{N}^{Ai}{s}_{N-1}^{Ai})/2$. In the absence of errors, stabilizer measurements remain unchanged between cycles, resulting in syndrome elements of zero. If a stabilizer measurement outcome changes between cycles, it signals an error, causing the corresponding syndrome element to flip, that is, change from 0 to 1, or vice versa, in the subsequent cycle.

Logical operations are performed using code deformations^17^. In our experiment, we perform an *X*-type lattice split on a distance-three surface-code logical qubit. We define the logical-qubit *X* and *Z* Pauli operators as the products of respective Pauli operators of the middle column ${\hat{X}}_{{{\rm{L}}}}={\hat{X}}_{{{\rm{D2}}}}{\hat{X}}_{{{\rm{D5}}}}{\hat{X}}_{{{\rm{D8}}}}$ and middle row ${\hat{Z}}_{{{\rm{L}}}}={\hat{Z}}_{{{\rm{D4}}}}{\hat{Z}}_{{{\rm{D5}}}}{\hat{Z}}_{{{\rm{D6}}}}$ of data qubits, as indicated in Fig. 1a. After *m* + 1 = 4 cycles of *X*-type stabilizer measurements interleaved with *m* = 3 cycles of *Z*-type stabilizer measurements, we read out the middle column of data qubits (D2, D5 and D8) in the *Z* basis. In addition, we stop any further *X*-type stabilizer measurements along the splitting boundary, which, in the case of the two repetition-code qubits, amounts to stopping all *X*-type syndrome extractions. This code deformation step is illustrated in Fig. 1b. The remaining data qubits D1, D3, D4, D6, D7 and D9 and *Z* stabilizers form two bit-flip repetition codes, as shown in Fig. 1c. After the split, we execute *n* = 2 cycles of *Z*-type stabilizer measurements of the bit-flip codes. The chosen values of *m* and *n* allow us to isolate the effects of state preparation, split and readout operations. We define the logical-qubit operators associated with the bit-flip codes as ${\hat{Z}}_{{{\rm{L1}}}}={\hat{Z}}_{{{\rm{D4}}}},{\hat{X}}_{{{\rm{L1}}}}={\hat{X}}_{{{\rm{D1}}}}{\hat{X}}_{{{\rm{D4}}}}{\hat{X}}_{{{\rm{D7}}}}$, ${\hat{Z}}_{{{\rm{L2}}}}={\hat{Z}}_{{{\rm{D6}}}},{\hat{X}}_{{{\rm{L1}}}}={\hat{X}}_{{{\rm{D3}}}}{\hat{X}}_{{{\rm{D6}}}}{\hat{X}}_{{{\rm{D9}}}}$.

As a result of the split operation, the single logical degree of freedom of the distance-three surface code is transformed into two distinct degrees of freedom. The new additional degree of freedom is associated with the *X*-type stabilizers, which are no longer measured, and is used to store logical information. Specifically, the product of the four *X*-type stabilizers corresponds to the product of the $\hat{X}$ operators of the remaining data qubits, which equals ${\hat{X}}_{{{\rm{L1}}}}{\hat{X}}_{{{\rm{L2}}}}$. For the $\hat{Z}$ operators, we can similarly obtain ${\hat{Z}}_{{{\rm{L1}}}}{\hat{Z}}_{{{\rm{L2}}}}={\hat{Z}}_{{{\rm{L}}}}{z}_{{{\rm{D5}}}}$, where *z*_D5_ is the measurement outcome of the central data qubit. Similar to quantum teleportation^23^, the entangled state of the logical qubits depends on the readout outcomes of the auxiliary degrees of freedom used to facilitate the entanglement.

Throughout this Article, we consider a deterministic version of the split operation. To that end, we apply a Pauli-frame update to the bit-flip-encoded logical qubits. If the final outcome of *s*^X2^*s*^X4^ (*s*^X1^*s*^X3^) is −1, we apply a virtual *Z* gate to the first (second) logical qubit. Similarly, if *z*_D5_ = −1, we apply a virtual *X* gate to the second logical qubit. The virtual gates are performed by flipping the logical qubit observable outcomes in postprocessing. The experimental sequence, together with the Pauli-frame update, is shown in Fig. 1d. In addition, the syndrome definition must be modified to include the readout outcome of the measured data qubits (see Supplementary Section I for details).

In the logical subspace, the Pauli-frame-updated *X*-type split operation maps a single qubit to two qubits. The mapping rule is that of a Hadamard-transformed fanout gate, that is, a CNOT gate, where the target qubit is initialized in the $| 0\rangle$ state^24^

1 $$\alpha | +\rangle +\beta | -\rangle \to \alpha |\!++\rangle +\beta |\!--\rangle .$$

For our experiments, we use a device similar to the one described in ref. ^7^. This device consists of 17 flux-tunable transmon qubits with a connectivity designed for implementing a distance-three surface code^25^. The qubits are controlled via individual charge lines (shown in pink) for single-qubit gates and flux lines (green) for two-qubit gates. The device features readout circuits, each consisting of readout-resonator–Purcell-filter pairs (red and blue) coupled to shared feedlines (purple) for frequency-multiplexed readout^26^ (see Fig. 1e for a false-coloured micrograph). The implementations of single-qubit gates, two-qubit gates and readout are described in the Methods. We do not reset qubits after their readout, the implications of which are discussed in refs. ^7,27^.

Using randomized benchmarking and interleaved randomized benchmarking, we measure average single-qubit and two-qubit gate errors of (0.09 ± 0.05)% and (2.2 ± 1.7)%. The average two-state and three-state readout assignment fidelity is 98.5% and 97.5%, respectively. For an integral error metric, we execute a state preservation experiment in the distance-three surface code, obtaining an average syndrome element value of 0.182 for weight-four syndromes and 0.114 for weight-two syndromes (see Supplementary Section II for details). Cumulative distribution functions of the individual gate and readout errors are shown in Fig. 1f together with the average syndrome elements.

**Extended Data Fig. 1:**
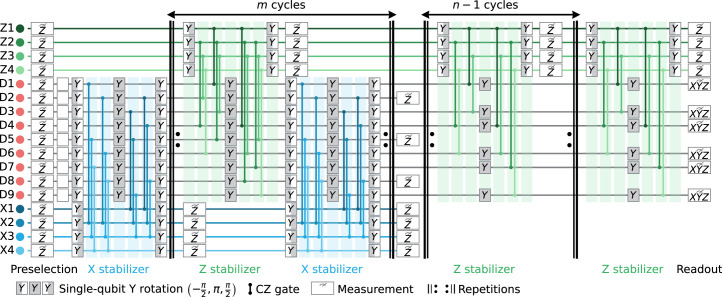
Experimental sequence of the lattice-split operation. Measurements are performed in the *Z* basis, except for the final logical quantum state tomography performed on the data qubits constituting the two repetition-code qubits, and indicated by *XYZ*. Empty boxes stand for single-qubit gates during state preparation.

We use a stabilizer measurement circuit that is fault-tolerant against circuit-level noise (Extended Data Fig. 1), described in detail in Methods. Assuming that only one error occurs—whether during a single-qubit gate, a two-qubit gate, qubit initialization or readout—the effect of that error on the logical-qubit observables can be correctly identified using the syndrome elements of the distance-three surface code^28^. *Z*-type stabilizer measurements are performed throughout the entire circuit, including preparation of a *Z*-basis cardinal state of the distance-three surface code, the split operation and the readout of *Z* observables. As a result of the fault-tolerant nature of lattice surgery, the *Z*_L1_ and *Z*_L2_ observables are protected from any single error. However, for the bit-flip repetition codes, *X*-type stabilizers are not measured, making it impossible to track errors affecting the *X*_L1_ and *X*_L2_ observables after the split operation.

## Logical Bell state preparation with a lattice split

**Fig. 2:**
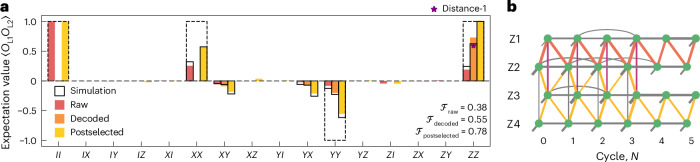
Characterization of the lattice-split operation executed on the state ${| 0\rangle }_{{{\rm{L}}}}$. **a**, Expectation values of the repetition-code logical-qubit-operator pairs after the split operation. Raw (red) and syndrome-postselected values (yellow) are shown for all 15 logical two-qubit observables. MWPM-decoded values (orange) are shown for *X*_L1_*Y*_L2_, *Y*_L1_*X*_L2_, *Y*_L1_*Y*_L2_ and *Z*_L1_*Z*_L2_. We mark (star) an experimentally extracted *Z*_L1_*Z*_L2_ value of a distance-one experiment for comparison. Dashed wireframes indicate ideal values; solid wireframes show simulation results. Logical-qubit indices are omitted for brevity. **b**, Experimentally extracted decoder matching graph for *Z*-syndromes. Nodes stand for individual syndrome measurements. Each row corresponds to one stabilizer operator in all cycles. Edges represent errors that cause the connected syndrome elements to flip. Thicker edges denote higher error probability. Red (orange) edges correspond to errors which flip the *Z*_L1_ (*Z*_L2_) observable, and purple edges correspond to errors which flip both repetition-code observables. Grey edges represent errors that do not affect the logical observables. See the main text for details.

To create a Bell state between two logical qubits using lattice surgery, we initialize the logical surface-code qubit in the ${| 0\rangle }_{{{\rm{L}}}}$ state. Ideally, a lattice-split operation, as described by equation (1), yields the two-qubit observables *X*_L1_*X*_L2_ = +1, *Z*_L1_*Z*_L2_ = +1 and *Y*_L1_*Y*_L2_ = − 1. We experimentally evaluate the logical two-qubit observables by measuring the product of the final data-qubit states of each logical repetition-code qubit. The experimental results agree well with simulations for the raw (red), error-corrected (orange) and error-detected (yellow) datasets (Fig. 2a). The observed systematic deviations of our data from the ideal values (dashed wireframes) are well captured by our model, as discussed in more detail below. We also note that we reject leakage events (Methods), retaining 76.8% of the 57,456 experimental runs.

As a consequence of the bit-flip fault tolerance of our experiment, we are able to decode *Z*-type syndrome data using a minimum-weight perfect matching (MWPM) decoder^29,30^. The matching graph is constructed by calculating weights based on correlations between syndrome measurements^31^, accounting for higher-weight error mechanisms^32^. For this experiment, the matching graph consists of four times six nodes corresponding to the extracted syndromes. Edges in the graph represent possible errors. We observe no statistically significant correlations between syndromes of the first bit-flip code ${\sigma }_{N}^{\rm{Z1}},{\sigma }_{N}^{\rm{Z2}}$ and the second bit-flip code ${\sigma }_{N}^{\rm{Z3}},{\sigma }_{N}^{\rm{Z4}}$ after the split operation (*N* = 3). We interpret this as the absence of correlated errors between the two distance-three repetition codes, highlighting the potential for independent state preservation in each logical qubit (Fig. 2b).

When we account for bit-flip errors in postprocessing, the expectation value of the *Z*_L1_*Z*_L2_ observable increases from its raw value of 0.189(5) to 0.730(3) and decreases for *Y*_L1_*Y*_L2_ from −0.082(5) to −0.199(5). While we observe a significant increase in the fault-tolerantly extracted expectation value of *Z*_L1_*Z*_L2_, the more modest improvement for *Y*_L1_*Y*_L2_ can be attributed to its susceptibility to non-correctable phase-flip errors (Fig. 2a). Performing quantum error correction, we improve the Bell state fidelity from *F*_raw_ = 0.382(2) to *F*_dec_ = 0.546(2). More details on decoding are provided in Supplementary Section III.

In error detection mode, we postselect on experimental runs where none of the syndrome measurements indicates an error. In this case, for the fault-tolerantly measured *Z*_L1_*Z*_L2_ observable, the postselected expectation value improves significantly to 0.998(1), approaching the ideal value of 1. By contrast, the *X*_L1_*X*_L2_ observable shows only a small increase from 0.255(5) to 0.56(2), and the *Y*_L1_*Y*_L2_ observable a decrease from −0.082(5) to −0.55(2), again due to phase-flip errors remaining undetectable after the lattice-split operation (Fig. 2a). The fraction of retained experimental runs after postselection is 6.3% for the *X*_L1_*X*_L2_ and *Y*_L1_*Y*_L2_ observables, and 5.5% for the *Z*_L1_*Z*_L2_ observable. The Bell state fidelity for the postselected dataset is *F*_postselected_ = 0.780(6) and is mostly limited by the non-fault-tolerantly extracted two-qubit observables.

In addition to the expected non-zero two-qubit observables, we also observe non-zero values for *X*_L1_*Y*_L2_ and *Y*_L1_*X*_L2_, which we attribute to a coherent phase shift of approximately 0.11π on one of the logical qubits. The observed phase shift is consistent with an AC-Stark shift on data qubit D1 induced by a mid-circuit measurement of a neighbouring data qubit. A detailed discussion of this phase error is provided in Supplementary Section IV.

We compare the measured expectation values with simulations of the experimental gate sequence, performed using Monte Carlo sampling of the 17-qubit wavefunction. The error model includes single-qubit and two-qubit depolarization channels, based on independently measured gate errors, and incorporates a coherent phase shift on data qubit D1 during the mid-circuit data qubit readout. The simulated two-qubit observables, shown as wireframes in Fig. 2a, agree well with the measured values and reproduce the effect of the experimentally observed coherent error. We find a norm-based fidelity^33^ between the decoded experimentally determined and simulated logical state density matrices of ${{{\mathcal{F}}}}_{2}=0.953(3)$. We discuss the details of the simulation and provide a breakdown of the simulated logical error probability for ${\hat{Z}}_{{{\rm{L1}}}}{\hat{Z}}_{{{\rm{L2}}}}$ into various error sources in Supplementary Section V.

To highlight the effectiveness of our bit-flip error correction scheme, we also perform a non-encoded variant of the split experiment using three data qubits, two *X*-type auxiliary qubits and *m* + 1 = 4 cycles of *X*-syndrome extraction, similar to the distance-three implementation. Despite the increased circuit complexity of the error-corrected code it shows an average ${\hat{Z}}_{{{\rm{L1}}}}{\hat{Z}}_{{{\rm{L2}}}}$ expectation value of 0.730(3), which is significantly closer to the ideal value of unity than 0.591(8) for the non-error-corrected variant (see star in Fig. 2a). Details of the non-error-corrected implementation can be found in Supplementary Section VI.

## Splitting arbitrary logical states

**Fig. 3:**
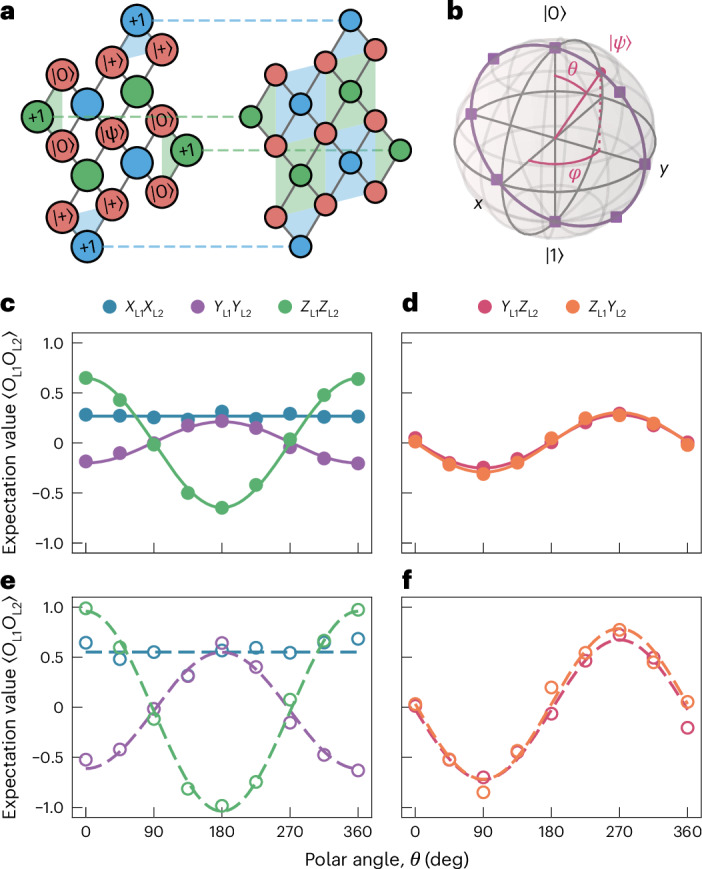
Lattice split of arbitrary states. **a**, Illustration of the arbitrary state preparation sequence for the distance-three surface code (see the text for details). **b**, Parametrization of the arbitrary state in the *yz* plane by the polar angle *θ*. Filled purple squares in the *yz* plane indicate state preparations used as input states for the lattice-split operation. **c**–**f**, Non-zero two-qubit observables versus polar angle *θ* in the *yz* plane for decoded (**c** and **d**) and postselected (**e** and **f**) observables. **c**,**e**, *X*_L1_*X*_L2_, *Y*_L1_*Y*_L2_ and *Z*_L1_*Z*_L2_ observables. **d**,**f**, *Y*_L1_*Z*_L2_ and *Z*_L1_*Y*_L2_ observables. The straight solid and dashed blue lines represent the average expectation value of *X*_L1_*X*_L2_, while the other solid and dashed lines correspond to sinusoidal fits of the *Y*_L1_*Y*_L2_, *Z*_L1_*Z*_L2_, *Z*_L1_*Y*_L2_ and *Y*_L1_*Z*_L2_ observable expectation values.

The lattice split is a quantum operation acting on an encoded qubit. To characterize its action on different initial logical states, we prepare arbitrary input states of the distance-three surface-code logical qubit via state injection^34,35^. We initialize the central data qubit (D5) in the target state $| {\psi} \rangle$, and the other data qubits in either the $| +\rangle$ or $| 0\rangle$ states, such that the weight-two stabilizers of the surface code are well defined. In Fig. 3a, the initial states of the data qubits are shown with the labels in the red circles. Stabilizers that are well defined are indicated by coloured plaquettes (blue or green). The labels in the blue and green circles correspond to the initial values of the stabilizers for this particular initialization of the data qubits. After the first error correction cycle, all stabilizers are in a well-defined state, indicated by filled plaquettes in Fig. 3a. The dashed lines between stabilizers indicate valid syndromes that can be used to detect errors.

Although this procedure is not fully fault-tolerant, it allows us to postselect on runs where no errors are detected by the weight-two stabilizers after the first stabilizer measurement cycle. Retaining 48% of the data on average, we prepare arbitrary logical states with an average postselected fidelity of 97.0% (see Supplementary Section VII for details). This scheme does not directly scale to larger codes; however, it can be adapted to a protocol that prepares noisy magic states, whose fidelity can be fault-tolerantly increased using distillation protocols^36^.

We evaluate the action of the lattice-split operation on arbitrary logical states in the *yz* plane of the Bloch sphere, parameterized by the polar angle *θ* with a fixed value of the azimuthal angle *φ* = 90° (Fig. 3b). We characterize the final state of the bit-flip codes using logical state tomography. Experimental runs with errors detected in the first round of stabilizer measurements are discarded.

For the polar angle *θ* = 0, the initial state is ${| 0\rangle }_{{{\rm{L}}}}$, and the outcomes are in line with the logical Bell state. As a function of polar angle *θ*, the *X*_L1_*X*_L2_ observable remains close to constant, independent of the initially prepared state, while the logical *Z*_L1_*Z*_L2_, *Y*_L1_*Y*_L2_, *Y*_L1_*Z*_L2_ and *Z*_L1_*Y*_L2_ observables show sinusoidal dependencies on *θ*, as expected from equation (1). We observe the same qualitative features when performing either error correction or postselecting on runs where no errors were detected. However, for postselection the contrast is higher for all observables. The highest contrast of 0.646(5) for the error-corrected observable and 0.97(2) for the postselected observable is obtained for *Z*_L1_*Z*_L2_, consistent with the fault-tolerant nature of the protocol. The *X*_L1_*X*_L2_ observable expectation values are independent of *θ*, showing an average value of 0.267(9) for the error-corrected and 0.55(3) for the postselected outcomes. The contrasts for *Y*_L1_*Y*_L2_, *Y*_L1_*Z*_L2_ and *Z*_L1_*Y*_L2_ also show a reduced magnitude. The expectation values of the indicated two-qubit observables evaluated with error correction are shown in Fig. 3c,d, and with postselection on no detected errors in Fig. 3e,f.

To further characterize the split operation acting on the logical qubit, we perform logical quantum process tomography^15,37^. By preparing the distance-three surface code in eigenstates of the ${\hat{X}}_{{{\rm{L}}}}$, ${\hat{Y}}_{{{\rm{L}}}}$ and ${\hat{Z}}_{{{\rm{L}}}}$ operators using the approach described above, applying the lattice-split operation and performing a tomographic readout with nine combinations of logical single-qubit observables, we reconstruct the process map.

**Fig. 4:**
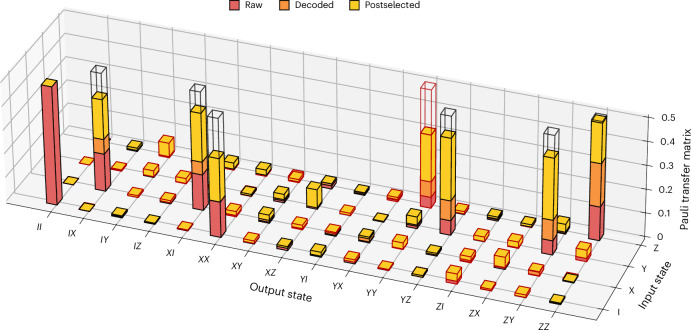
Pauli transfer matrix (PTM) of the split operation reconstructed from raw (red), decoded (orange) and postselected (yellow) logical observable outcomes. The input state operators refer to the distance-three surface-code observable *O*_L_, and the output state operators refer to joint observables of the two bit-flip repetition codes *O*_L1_*O*_L2_. The height of each coloured bar or wireframe indicates the absolute value of the corresponding PTM element. Wireframe colour indicates the sign of the PTM entry (black for positive, red for negative). The empty wireframes show the PTM entries expected for an ideal split operation.

The transformation of a single distance-three surface-code logical qubit into two distance-three bit-flip repetition-code logical qubits after the split is described by a 4 × 16 process matrix. For the reconstruction of the non-square process matrix, we follow the conventions described in ref. ^38^. We reconstruct the Choi matrix with the positive semi-definite constraint, ensuring complete positivity of the process map. Reconstruction is performed for three sets of observables: raw, decoded and postselected on no detected errors in the entire run. The resulting process fidelity for the raw outcome is 0.310(4). Decoding improves the process fidelity to 0.442(4). Postselection yields a process fidelity of 0.781(11), while retaining only 3.3% of the experimental runs. In Fig. 4, we show the Pauli transfer matrix representations of the raw (red), decoded (orange) and postselected (yellow) process maps, with black (red) wireframes around entries indicating positive (negative) matrix values. Furthermore, in the analysis, we correct for the coherent phase rotation of 0.11π observed in the first bit-flip repetition-code qubit (see Supplementary Section IV for details). The eight entries that are expected to take non-zero values are highlighted by empty wireframes with the ideal absolute value of 0.5. We find good qualitative agreement between the reconstructed Pauli transfer matrix and the results of our stabilizer simulation (see Supplementary Section V for details).

In this work, we have experimentally demonstrated a lattice-split operation on a distance-three surface code, exploring lattice surgery for entangling logical qubits. In addition, our experiments highlight the utility of logical process tomography performed on elementary error-corrected operations to identify and mitigate effective errors at the logical level. We anticipate that future experiments implementing lattice surgery protocols with two distance-three (or larger) surface codes will achieve fault tolerance not only for bit-flip errors but also for phase-flip errors, further reducing both coherent and incoherent error rates. The characterization techniques explored in this work will be valuable for future realizations of lattice surgery.

## Methods

### Idling and single-qubit gates

During idling and single-qubit gate operations, we nominally apply an integer flux in units of the flux quantum to the asymmetric superconducting quantum interference devices (SQUIDs) of the auxiliary qubits, and half-integer flux to the SQUIDs of the data qubits. This biases the qubits at the upper and lower first-order flux-insensitive frequencies, respectively. At these bias points, we measure energy relaxation times *T*_1_ between 24 μs and 78 μs and coherence times ${T}_{2}^{E}$ ranging from 12.4 to 138.9 μs. Due to a two-level system defect at the upper flux-insensitive point of X3, this qubit was instead operated at its lower flux-insensitive point. For single-qubit gates, we apply 48-ns derivative removal by adiabatic gate (DRAG) microwave pulses^39,40^ via a coplanar waveguide capacitively coupled to the qubit island. We compensate for microwave crosstalk as described in ref. ^7^ for qubit pairs where the crosstalk has been shown to adversely affect gate fidelity.

### Qutrit readout and leakage rejection

We measure the qubit states using dispersive single-shot readout via a capacitively coupled readout circuit^41^. The readout is executed with Gaussian-filtered square microwave pulses applied to the feedline. The pulse frequency is optimized to maximize the separation of the complex scattering amplitudes of the readout signals in the IQ plane for the prepared transmon states. After choosing the readout frequency, the amplitude and duration of the pulse are tuned for maximum single-shot assignment fidelity, resulting in durations ranging from 175 to 300 ns across the 17 qubits. By measuring the response of the transmitted readout signal in the time domain, with the qubit prepared in its ground, first and second excited states, we extract optimal weights for the qubit readout^42^. In addition, for qubit X3, we use flux-pulse-assisted readout^43^, reducing the qubit-readout-resonator detuning and increasing the effective coupling of the readout resonator mode to the readout feedline for improved performance.

In our current implementation, we do not correct for leakage errors. We discard all runs of the experiment where a qubit is detected in a leaked state. In future experiments, leakage reduction units^44,45^ can be used to convert leakage errors into Pauli errors, which has been shown to improve error correction performance for codes with distance larger than three^14^.

### Two-qubit gates

We implement two-qubit gates by leveraging the coherent evolution of neighbouring qubits between the $| 11\rangle$ and $| 02\rangle$ states^46^. This is achieved using a static capacitive coupling mediated by a coplanar waveguide resonator, with fast flux pulses applied to the SQUIDs of both qubits to bring the states into resonance at a desired interaction frequency within their tunability range. To mitigate distortions of the control signals, we use infinite impulse response and finite impulse response filters^47^, along with flux crosstalk compensation as described in ref. ^7^. We operate the gate using net-zero pulses^48^, with the duration between the two halves of the pulses fine-tuned to adjust the conditional phase^49^. Using defect-mode swap spectroscopy^50^, we scan for the presence of two-level defects and select interaction frequencies that minimize energy loss to these defects while maintaining sufficient spectral distance from transition frequencies of neighbouring qubits to avoid spectator errors^51^. The average duration of the CZ gate is 101.5 ns. Additional 20 ns buffers are added at the beginning and the end of the net-zero pulse. We perform interleaved randomized benchmarking on each two-qubit gate individually to characterize the performance (Fig. 1f), rather than benchmarking gates in parallel, as the set of simultaneously active gates differs before and after the lattice split.

### Gate sequence of lattice split operation

The stabilizer measurements are realized using a gate sequence similar to the one in ref. ^7^, consisting of single-qubit π/2 and π rotations, CZ gates and mid-circuit auxiliary-qubit measurements. Within each cycle, active stabilizers of the same type are measured simultaneously. The gate sequence for a stabilizer measurement consists of eight time steps, in which all gates within the same step are executed in parallel. The first and seventh steps are π/2 rotations on the auxiliary qubits. For *X*-type stabilizers, π/2 pulses are also applied to the data qubits. The second, third, fifth and sixth steps contain two-qubit gates. The order of the two-qubit gates is chosen to maintain fault tolerance^28^. Gates involving inactive qubits, that is, *X*-type auxiliary qubits or data qubits in the middle row after the split, are not executed. We incorporate dynamical decoupling by applying echo pulses to the data qubits in the fourth step, mitigating low-frequency noise on the flux control lines and spectator errors^51^. Finally, the eighth step is the auxiliary-qubit readout. During surface-code cycles, we use a pipelined gate scheduling^25^: a new stabilizer measurement begins before the readout step of the other stabilizer type completes. The duration between the start of successive stabilizer measurement cycles of the same type is 1.66 μs. Data qubit readout—both mid-circuit during the code deformation and in the final measurement—is performed in parallel with the auxiliary-qubit readout. The gate sequence for the lattice-split experiment includes a preselection readout, state preparation, repeated stabilizer measurements and final data-qubit readout (Extended Data Fig. 1). The preselection readout is used to discard runs in which any qubit is detected in the excited or leaked state at the beginning of the sequence. No state preparation pulses are required for the logical ${| 0\rangle }_{{{\rm{L}}}}$ state; for the arbitrary state split, single-qubit gates are executed according to the states shown in Fig. 3b.

The purpose of the echo pulses in the middle of the stabilizer measurement gate sequence is to cancel the effect of static interactions that couple through the data-qubit *Z* operators. Such interactions include slow frequency drifts, which can be caused by two-level fluctuators, as well as off-resonant interactions between data qubits and neighbouring auxiliary qubits during idling periods^51^. As a side effect of the echo pulses, during each *Z*-type stabilizer measurement, the data qubits undergo a *Y* rotation due to the echo pulse. In the *X*-type stabilizer measurements, the echo rotation is compensated by the initial and final π/2 rotations. Rotations on the data qubits are corrected in postprocessing of their readout outcomes.

## Online content

Any methods, additional references, Nature Portfolio reporting summaries, source data, extended data, supplementary information, acknowledgements, peer review information; details of author contributions and competing interests; and statements of data and code availability are available at 10.1038/s41567-025-03090-6.

## Supplementary information

Supplementary Information Supplementary Figs. 1–9 and Discussion.

Supplementary Data 1 Source data for Supplementary figures.

## Data Availability

The data generated in this study have been deposited in the ETH Zurich repository for research data and are publicly available at 10.3929/ethz-c-000783470.

## Code availability

The code used for data analysis is available from the corresponding authors upon request.
