# Supplementary material for: Lattice surgery realized on two distance-three repetition codes with superconducting qubits
Source: Nat Phys. 2026 Jan 30;22(2):189–94. doi: 10.1038/s41567-025-03090-6 (PMC12904781; doi:10.1038/s41567-025-03090-6)
Supplement: Supplementary file 1 — Supplementary Figs. 1–9 and Discussion. [file 41567_2025_3090_MOESM1_ESM.pdf]

nature physics

Supplementary information

https://doi.org/10.1038/s41567-025-03090-6

Lattice surgery realized on two distance-three repetition codes with superconducting qubits

In the format provided by the authors and unedited

CONTENTS

I. Pauli Frame Update 1

II. Performance of Logical Qubit Idling 1

III. Error Correction for Tomographic Readouts 4

IV. Coherent Rotation Quantification and Correction 5

V. Simulation 6

VI. Distance-One Implementation of Bell-State Preparation Protocol 9

VII. Arbitrary State Preparation and Tomography of a Logical Qubit 10

I. PAULI FRAME UPDATE

During a code deformation, data qubits can be added to or removed from stabilizers. For a code deformation to be fault-tolerant, any data qubit added to an existing X-type (Z-type) stabilizer must be initialized in an eigenstate of the $\hat{X}$ ($\hat{Z}$) operator [1]. In the absence of errors, the stabilizer measurement outcome after adding the new data qubit can be predicted from the previous cycle's outcome and the chosen initial state of the new qubit. Conversely, removing a data qubit from a stabilizer while acquiring a syndrome element from that stabilizer requires the data qubit to be read out in the stabilizer basis. Depending on the initialization state or the readout outcome, the code deformation can result in a flip of the stabilizer. For our split experiment, the code deformation involves reading out three data qubits. The stabilizers $\hat{S}^{\mathrm{Z2}}$ and $\hat{S}^{\mathrm{Z3}}$ are defined both before and after the code deformation, but their data-qubit composition changes during the X-type split. To accommodate the changing stabilizer compositions, we use a modified definition of syndrome elements in error correction cycle $N = 3$ by multiplying the stabilizer values with the mid-circuit data-qubit readout outcomes z_{D2}, z_{D5}, and z_{D8}:

$$\begin{aligned}\sigma_3^{\mathrm{Z2}} &= (1 - s_3^{\mathrm{Z2}} s_2^{\mathrm{Z2}} z_{\mathrm{D5}} z_{\mathrm{D8}})/2,\\ \sigma_3^{\mathrm{Z3}} &= (1 - s_3^{\mathrm{Z3}} s_2^{\mathrm{Z3}} z_{\mathrm{D2}} z_{\mathrm{D5}})/2.\end{aligned} \tag{S1}$$

To obtain deterministic logical observable outcomes, we apply a Pauli-frame update to the logical-qubit operators. As a post-processing operation, the Pauli-frame update, shown in Fig. 1d, can be expressed as

$$\begin{aligned}\hat{X}_{\mathrm{L1}} &\to \hat{X}_{\mathrm{L1}} s_3^{\mathrm{X2}} s_3^{\mathrm{X4}},\\ \hat{X}_{\mathrm{L2}} &\to \hat{X}_{\mathrm{L2}} s_3^{\mathrm{X1}} s_3^{\mathrm{X3}},\\ \hat{Z}_{\mathrm{L1}} &\to \hat{Z}_{\mathrm{L1}},\\ \hat{Z}_{\mathrm{L2}} &\to \hat{Z}_{\mathrm{L2}} z_{\mathrm{D5}}.\end{aligned} \tag{S2}$$

The transformation involves the X-type stabilizer outcomes, which are measured $m + 1 = 4$ times before the split. In the absence of errors, the syndrome elements of the same stabilizer are identical across cycles. However, errors occurring before the last cycle will flip the stabilizer measurement outcomes along with the logical observables. To obtain the best estimate of the final stabilizer outcome we use $s_3^{\mathrm{X}i}$ for the Pauli-frame update.

To motivate the modification of the logical-qubit definitions given by Eq. (S2), we consider the definitions of the X-type logical operators $\hat{X}_{\mathrm{L}}$, $\hat{X}_{\mathrm{L1}}$, and $\hat{X}_{\mathrm{L2}}$. For a surface code, these definitions are equivalent up to a product of stabilizer operators (Fig. S1a and b). Specifically,

$$\begin{aligned}\hat{X}_{\mathrm{L}} \hat{X}_{\mathrm{L1}} &= \hat{S}^{\mathrm{X2}} \hat{S}^{\mathrm{X4}},\\ \hat{X}_{\mathrm{L}} \hat{X}_{\mathrm{L2}} &= \hat{S}^{\mathrm{X1}} \hat{S}^{\mathrm{X3}}.\end{aligned} \tag{S3}$$

These relations hold until the middle column of data qubits is read out in the Z basis, after which the definitions of $\hat{X}_{\mathrm{L}}$ and $\hat{S}^{\mathrm{X}i}$ no longer refer to quantities that can be evaluated. By substituting the operators $\hat{S}^{\mathrm{X}i}$ with their final measured outcomes $s_3^{\mathrm{X}i}$, we obtain the Pauli-frame updates for the X observables in (S2).

For the Z-type logical observables, shown in Fig. S1c, we have

$$\hat{Z}_{\mathrm{L}} = \hat{Z}_{\mathrm{D4}} \hat{Z}_{\mathrm{D5}} \hat{Z}_{\mathrm{D6}}. \tag{S4}$$

After reading out D5 in the Z basis, the operator $\hat{Z}_{\mathrm{D5}}$ can be replaced by the measurement outcome z_{D5}. To ensure the deterministic relation $\hat{Z}_{\mathrm{L}} = \hat{Z}_{\mathrm{L1}} \hat{Z}_{\mathrm{L2}}$, the readout outcome of D5 must be included in the definition of either logical operator $\hat{Z}_{\mathrm{L1}}$ or $\hat{Z}_{\mathrm{L2}}$. We choose to append it to $\hat{Z}_{\mathrm{L2}}$. The final expressions for the Pauli-frame-updated Z observables of the bit-flip codes are

$$\begin{aligned}\hat{Z}_{\mathrm{L1}} &= \hat{Z}_{\mathrm{D4}},\\ \hat{Z}_{\mathrm{L2}} &= \hat{Z}_{\mathrm{D6}} z_{\mathrm{D5}}.\end{aligned} \tag{S5}$$

Due to the appended measurement outcome z_{D5}, the errors corresponding to edges between Z2 and Z3 in the decoder graph in Fig. 2b flip the Z_{L2} observable.

II. PERFORMANCE OF LOGICAL QUBIT IDLING

We characterize the performance of our distance-three surface-code qubit using the state preservation experiment of Ref. 2. As an integral characteristic of the physical

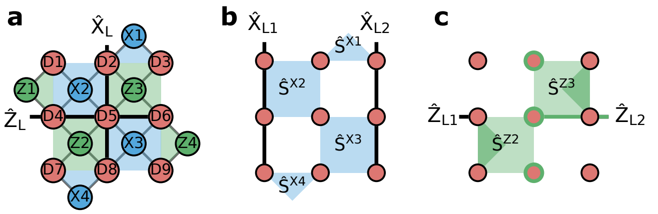

FIG. S1: Definitions of logical-qubit operators of the distance-three surface code and the bit-flip repetition codes and their relation to data-qubit readout and stabilizers. The red circles indicate data qubits, green circles Z-type and blue circles X-type auxiliary qubits. **a** Naming scheme of data and auxiliary qubits, as well as the definition of the $\hat{Z}_{\mathrm{L}}$ and $\hat{X}_{\mathrm{L}}$ for the distance-three surface code. **b** Definition of X-type stabilizers and repetition-code X-type observables. Black vertical lines show strings of data qubits in the definitions of $\hat{X}_{\mathrm{L1}}$, and $\hat{X}_{\mathrm{L2}}$. For each X-type stabilizer, a light-blue rectangle illustrates which data qubits' parity is included in the stabilizer. **c** Definition of repetition-code Z-type logical observables. The black lines show the data qubits, whose $\hat{Z}$ operators are in the definition of the $\hat{Z}_{\mathrm{L1}}$ and $\hat{Z}_{\mathrm{L2}}$ logical-qubit operators. The green line extending from $\hat{Z}_{\mathrm{L2}}$ shows that the central data-qubit readout outcome is included in the Pauli-frame update for $\hat{Z}_{\mathrm{L2}}$. The light-green and dark-green plaquettes indicate the modified Z-type stabilizers before and after the split operation, respectively.

error rate, we determine the average syndrome values across Z-type and X-type stabilizers [3]. For the Z-type stabilizers, we prepare the data qubits in the $|0\rangle^{\otimes 9}$ and $\hat{X}_{\mathrm{L}}\,|0\rangle^{\otimes 9}$ states. For the X-type stabilizers, we prepare the data qubits in the $|+\rangle^{\otimes 9}$ and $\hat{Z}_{\mathrm{L}}\,|+\rangle^{\otimes 9}$ states. The cycle duration is 1.66 μs.

After initializing the data qubits, we perform 20 cycles of interleaved X-type and Z-type stabilizer measurements. The gate sequence is similar to the first half of the one used for the lattice-split experiment, shown in Fig. 5, but omits the final round of X-type stabilizer measurements. Finally, we read out the data qubits in the same basis as the initial state preparation.

The initial values of the stabilizers are defined by the initialized data-qubit states, while the final values of the stabilizers are determined from the data-qubit readout outcomes. The first syndrome elements are calculated as parities of the stabilizer values determined by state preparation and the outcome of the first cycle of stabilizer measurements. The final syndrome element is calculated as the parity of the data-qubit readout outcomes and the outcome of the last cycle of stabilizer measurements. All other syndrome elements, also referred to as "bulk" syndromes, are calculated as the parities of subsequent stabilizer measurement outcomes.

We discard runs where any readout has resulted in a leaked outcome. For weight-four stabilizers, we find an average syndrome element of 0.182, while for weight-two stabilizers it is 0.114. This is expected, as the four-qubit stabilizer syndrome extraction circuit is sensitive to errors in twice as many two-qubit gates. The initial and final syndromes are flipped by errors in fewer two-qubit gates, which explains the lower average value of these syndrome elements (0.075 and 0.125) compared to bulk syndrome elements (0.153). The average Z-type (X-type) syndrome elements are shown in Fig. S2a as a function of the stabilizer measurement cycle m with green (blue) circles for weight-two syndromes and squares for weight-four syndromes.

In addition to the differences between weight-two and weight-four syndrome elements, and bulk, initial, and final syndrome elements, bulk syndromes show a gradual increase in their mean values with cycle number. One effect contributing to this behavior is biased noise during auxiliary-qubit readout. When initializing the logical qubit in the Z (X) basis, the Z-type (X-type) stabilizers are initialized in the $+1$ state. Before and during the readout, auxiliary qubits are expected to remain in the ground state, which is not prone to decay errors. Without auxiliary-qubit reset, errors accumulating over cycles cause the auxiliary qubits to approach an asymptotic population of 0.5 and increase the impact of decay errors. Another effect that leads to the observed gradual increase of the syndrome elements is undetected data-qubit leakage accumulation. For the surface code (repetition codes), in the second cycle the average syndrome element is 0.13 (0.08), and in the second-to-last round it is 0.16 (0.11).

Similarly, we extract the mean syndrome elements for the bit-flip codes. In this case, we perform fault-tolerant preparation of the $\hat{Z}_{\mathrm{L1}}\hat{Z}_{\mathrm{L2}}$ eigenstates: the active data qubits D1, D3, D4, D6, D7, and D9 are initialized in the $|0\rangle^{\otimes 6}$, $\hat{X}_{\mathrm{L1}}\,|0\rangle^{\otimes 6}$, $\hat{X}_{\mathrm{L2}}\,|0\rangle^{\otimes 6}$, and $\hat{X}_{\mathrm{L1}}\hat{X}_{\mathrm{L2}}\,|0\rangle^{\otimes 6}$ states. We perform 20 cycles of syndrome extraction, with the same circuit as used in the second half of the split circuit (Fig. 5). Simultaneously with the auxiliary-qubit readout of the last cycle of syndrome extraction, we read out the data qubits in the Z basis. Similar to the case of the surface code, this yields 19 cycles of bulk syndromes calculated as parities of stabilizer measurement outcomes, and two extra syndromes from the fault-tolerant preparation and readout. We use the same cycle time of 1.66 μs as in the distance-three surface code. The average syndrome elements over both bit-flip codes is 0.095, and is shown as a function of the stabilizer measurement cycle m in Fig. S2b. The higher value of the average weight-two syndrome elements in the distance-three surface code compared to the repetition codes can be attributed to gate errors that occur during the X-type syndrome extraction circuit.

Next, we perform a state preservation experiment for the distance-three surface code with varying number of error correction cycles, as in Refs. 2, 4–6. The $\hat{X}_{\mathrm{L}}$ ($\hat{Z}_{\mathrm{L}}$) eigenstates are prepared fault-tolerantly, and after m interleaved cycles of X-type and Z-type stabilizer measurements, the data qubits are read out in the X (Z) basis. Additionally, we consider a dataset where the logical qubit

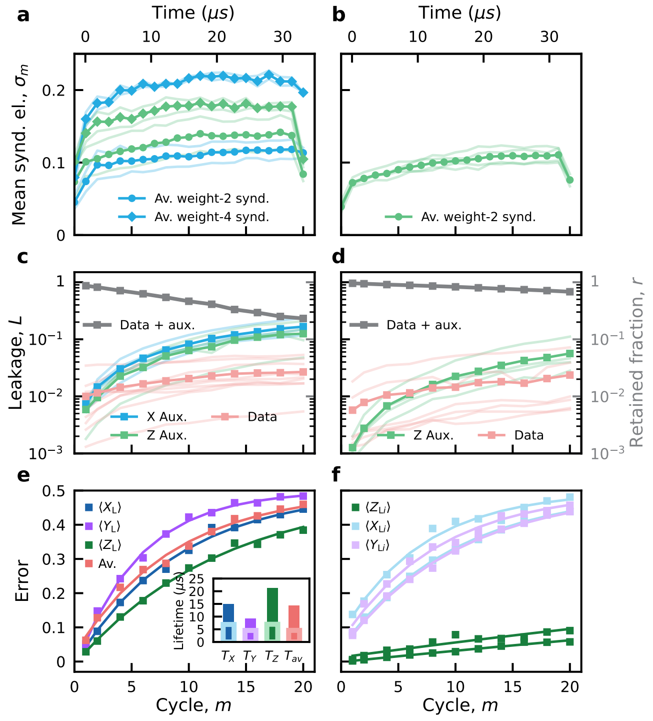

FIG. S2: Performance of surface-code and repetition-code experiments. **a** Mean syndrome elements of the surface-code experiment. The lighter green (blue) lines show Z-type (X-type) syndrome data, and the darker lines indicate the average over stabilizers with the same weight. **b** Mean syndrome elements for the repetition codes. The darker line shows the average over all Z-syndrome data. **c** and **d** Leakage and retention rates for the surface-code and repetition-code experiment. The darker red, blue and green lines indicate the average leakage of data, X-type auxiliary, and Z-type auxiliary qubits, respectively. The gray line indicates the retained fraction of experimental runs after having postselected on no leakage events. **e** (**f**) Logical error probability for the distance-3 surface code (bit-flip repetition code) qubit observables. Inset: extracted raw (wireframe) and decoded lifetimes for different observables of the distance-3 surface-code qubit.

is initialized non-fault-tolerantly using the arbitrary state preparation scheme, and logical state tomography is carried out to determine the final state after m interleaved cycles of X-type and Z-type stabilizer measurements. The initialization and readout of arbitrary logical states are described in Section VII.

For the bit-flip repetition codes, we perform the logical state preservation experiment with fault-tolerant preparation of the $\hat{Z}_{L1}\hat{Z}_{L2}$ eigenstates followed by m cycles of Z-type syndrome extraction and data-qubit readout in the Z basis. Additionally, we perform experiments preparing the data qubits in the $|+\rangle^{\otimes 6}$, $\hat{Z}_{L1}|+\rangle^{\otimes 6}$, $\hat{Z}_{L2}|+\rangle^{\otimes 6}$, and $\hat{Z}_{L1}\hat{Z}_{L2}|+\rangle^{\otimes 6}$ states, and final data qubit readout in the X basis for determining the error rate in the X observables. In this case, the preparation and readout are not fault-tolerant, due to missing X-type syndrome extraction. For determination of Y observable error rates we prepare separable $\hat{Y}_{L1}\hat{Y}_{L2}$ eigenstates. The active data qubits D1, D3, D4, D6, D7, and D9 are initialized in the $|+i++i+\rangle$, $\hat{Z}_{L1}|+i++i+\rangle$, $\hat{Z}_{L2}|+i++i+\rangle$, and $\hat{Z}_{L1}\hat{Z}_{L2}|+i++i+\rangle$ states. Here, $|i\rangle$ stands for the $+1$ eigenstate of the $\hat{Y}$ operator. The final data-qubit readout is performed in the Y basis for the middle row of data qubits (D4 and D6), and in the X basis for all other data qubits.

Again, we discard all experimental runs where any one of the qubits has been detected in the leaked state. The data qubits are measured only at the end of the gate sequence. We observe an increase in the average probability of data qubits yielding a leaked state with the number of error correction cycles m. This dependence is shown by the red curves in Fig. S2c and d. For the surface code, the data-qubit leakage probability shows signs of saturation, indicating a finite lifetime of the leaked state, defined as the average number of cycles a qubit remains outside the computational basis. By fitting exponential models to these dependencies, we obtain per-cycle leakage rates of $2.3(2)\times10^{-3}$ and an average leaked state lifetime of 14 ± 2 cycles for the surface code. For the repetition code, the per-cycle leakage rate is $1.2(4)\times10^{-3}$, while the leaked state lifetime exceeds the maximum number of cycles in the experiment and cannot be reliably determined.

The auxiliary qubits are read out every cycle. We investigate the average probability of an auxiliary qubit yielding at least one leaked state readout outcome during m cycles of error correction. In the evaluated range of up to $m = 20$, these dependencies are linear, showing a per-cycle leakage rate of $8.7(4)\times10^{-3}$ ($7.0(8)\times10^{-3}$) for X-type (Z-type) auxiliary qubits in the surface code, and $2.9(4)\times10^{-3}$ for the Z-type auxiliary qubits in the bit-flip repetition codes. The green (blue) curves in Fig. S2c and d show the average probability for Z-type (X-type) auxiliary qubits yielding at least one leaked state readout outcome in a state preservation experiment with a total of m cycles of error correction. The higher auxiliary-qubit leakage compared to data qubits is expected because auxiliary qubits temporarily populate a non-computational state during our CZ gate implementation (see Methods for details). Finally, after performing leakage rejection for experimental runs where at least one transmon readout yields the leaked state, we obtain an exponential dependence of retained runs on the number of error correction cycles. The number of experimental runs remaining after leakage rejection is indicated by the gray curves in Fig. S2c and d. Using an exponential model fit, we obtain a per-cycle retention probability of $r_c = 0.931$ for the surface code, which is slightly exceeding $r_c = 0.921$ cited in Ref. 2, and $r_c = 0.983$ for two simultaneously executed bit-flip repetition codes. For the latter, the data-qubit

and Z-type auxiliary-qubit leakage is reduced due to fewer gate operations contributing to leakage errors.

After leakage rejection, we use syndrome correlation analysis [7, 8] to obtain edge weights for the Z and X decoder graphs. The syndrome data are decoded separately for Z and X decoders using minimum-weight perfect matching (MWPM). The logical error probability increases with the number of cycles for both the X and Z observables. The green (blue) data points in Fig. S2e show the average error in the logical Z_{L} (X_{L}) observables calculated from the final data-qubit readout after correcting with the decoder output. The blue and green curves show exponential fits to the experimental results. From the fits, we obtain per-cycle error probability estimates of $\epsilon_Z = 0.078(2)$ for the Z_{L} observable and $\epsilon_X = 0.111(4)$ for the X_{L} observable.

Using the arbitrary state preparation scheme, we demonstrate the preservation of 26 different states on the Bloch sphere (see Section VII). The average error after m cycles of stabilizer measurements, as determined through logical state tomography, is shown in red in Fig. S2e (red data points). Similar to the $\hat{X}_{\mathrm{L}}$ and $\hat{Z}_{\mathrm{L}}$ observables, the error also increases with the number of cycles. From the exponential model fit, we obtain an average per-cycle error rate of $\epsilon_{\mathrm{av}} = 0.117(5)$. The Y_{L} observable eigenstates exhibit the largest errors, since they are maximally susceptible to both X and Z errors. The dependence of the error on the number of cycles for the Y_{L} observable is shown in Fig. S2e as the purple curve. The per-cycle error rate for the Y_{L} observable is $\epsilon_Y = 0.179(6)$. The ratios of the cycle time and and the per-cycle error probabilities yield the effective coherence times T_X, T_Y, T_Z, and T_{av}. The effective coherence times of the error-corrected logical qubits are significantly higher than those of non-corrected logical qubits for all prepared states. The comparison between error-corrected and non-error-corrected coherence times is shown in the inset of Fig. S2e.

For the repetition codes, decoding is only available for Z observables. We use a correlation analysis procedure to obtain a decoder graph for the Z syndromes, similar to the surface-code experiment. The logical error probability in $\hat{Z}_{\mathrm{L1}}$ ($\hat{Z}_{\mathrm{L2}}$) increases with the number of cycles m, starting with 0.4×10^{-2} (1.3×10^{-2}) after a single error correction cycle, and reaching 11.5×10^{-2} (18.1×10^{-2}) after 20 cycles. The green points in Fig. S2f show the error probabilities in the Z observable outcomes when decoding with MWPM. From an exponential fit, we obtain per-cycle error rates of $\epsilon_{\mathrm{Z1}} = 0.0068(3)$ and $\epsilon_{\mathrm{Z2}} = 0.0093(11)$ for the two logical qubits. The X and Y observable outcomes cannot be corrected for phase errors. Nevertheless, the raw (uncorrected) outcome values can be evaluated, and the errors in the uncorrected logical observables show similar exponential dependencies, as shown by the light blue and purple points in Fig. S2f. Exponential fits to the data yield per-cycle error rates $\epsilon_{X1} = 0.101(2)$, $\epsilon_{X2} = 0.140(8)$, $\epsilon_{Y1} = 0.099(2)$, and $\epsilon_{Y2} = 0.118(5)$. These error rates are noticeably worse than those for the Z observables but are only slightly better than the per-cycle error rates for the error-corrected X and Y observables in the distance-three surface code. This behavior is expected in the near-threshold regime, where increasing the code distance does neither significantly improve nor degrade the performance of the code.

III. ERROR CORRECTION FOR TOMOGRAPHIC READOUTS

For decoding, we assume a Pauli circuit noise model. In this model, during the Bell state preparation and the measurement of the $Z_{\mathrm{L1}}Z_{\mathrm{L2}}$ observable, all errors that flip the same combination of syndromes have the same effect on the logical observable. This property underpins the fault tolerance of the circuit.

Any error affects at most two X syndrome elements and at most two Z syndrome elements. Any Y error, i.e., an error that flips both X and Z syndrome elements, can be represented as two separate errors: one affecting only X syndromes and the other affecting only Z syndromes. These decomposed errors have the same impact on the observables as the original error. Leveraging this separability property, we decompose the decoding problem into two smaller subproblems: decoding bit-flip errors, also referred to as X errors, using Z syndrome data and decoding phase-flip (Z) errors using X syndrome data. This approach simplifies the decoding process, but it does so at the cost of disregarding correlations between X and Z errors.

The decoding problem can be formulated as finding the most likely combination of errors that matches the observed syndrome elements. This is achieved by using graph representations for the X and Z syndrome elements. In the syndrome graph, each syndrome element corresponds to a node, while edges represent error mechanisms that flip the two connected syndrome elements. To handle errors that flip only a single syndrome element, a special "boundary" node is introduced, which is not associated with any measured syndrome. Edges connected to the boundary node are referred to as boundary edges. Each edge is assigned a weight w, which is related to the probability p of the error mechanism causing a flip of the associated syndrome elements. The relation between weight and probability is $w = -\log(p/(1-p))$. Within this graph representation, the decoding problem reduces to solving the minimum-weight perfect matching (MWPM) problem, which we address using the blossom algorithm [9], implemented in the pymatching package [10].

For quantum state tomography, all edges in the Z (X) decoding graph are classified based on whether the associated errors flip the Z_{L1} or Z_{L2} (X_{L1} or X_{L2}) logical observables. Any two-logical-qubit observable can be expressed as a product of these four operators. Using this representation, along with the results of the MWPM algorithm, we determine whether a correction is required for the raw measurement outcome.

For the most likely X errors, the mapping between

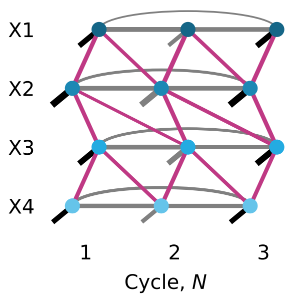

FIG. S3: Experimentally extracted decoder matching graph weights for X syndromes and their impact on logical observables. Gray edges correspond to errors that flip neither X_{L1} nor X_{L2}. Purple edges indicate errors that flip both X_{L1} and X_{L2}. Ambiguous edges associated with multiple errors with different effects on the logical observables are shown in black.

edges and their effects on the logical observables is shown in Fig. 2b. If the final readout of one of the logical qubits is performed outside the Z basis, the final set of syndrome elements, $\sigma_5^{\mathrm{Z}i}$, becomes non-deterministic, i.e., cannot be computed because the final stabilizer value is unavailable. In this case, the syndromes from the final cycle are not used.

For the X-type syndrome data, the circuit noise model yields three classes of edges: those corresponding to errors that do not flip any logical observables, those corresponding to errors that flip both $\hat{X}_{\mathrm{L1}}$ and $\hat{X}_{\mathrm{L2}}$, and ambiguous boundary edges. These edge classes are shown with different colors in Fig. S3 together with the experimentally extracted edge weights [8]. Ambiguous boundary edges arise due to the absence of X-type stabilizer data before the first round and after the split operation. Since there are no edges that are unambiguously associated with a flip of either $\hat{X}_{\mathrm{L1}}$ or $\hat{X}_{\mathrm{L2}}$, but not both, there are no phase-flip errors that can be corrected for the $X_{\mathrm{L1}}X_{\mathrm{L2}}$ and $Y_{\mathrm{L1}}Y_{\mathrm{L2}}$ observables. Therefore, for the Bell state, X syndrome decoding does not provide any improvement to the state fidelity. We note that the ambiguous edges in the decoding graph and the resulting lack of fault tolerance against phase-flip errors stem from our experiment encoding the two logical qubits in bit-flip repetition codes. An implementation on a larger device using, e.g., two distance-three surface codes instead would allow the decoding of both Z-type and X-type syndrome data, thereby enabling the correction of phase-flip errors.

For the observables $\hat{X}_{\mathrm{L1}}\hat{Y}_{\mathrm{L2}}$ and $\hat{Y}_{\mathrm{L1}}\hat{X}_{\mathrm{L2}}$ that take a non-zero value due to the coherent rotation error, the effect of the edges in the decoding graph depends on the nature of the coherent error. We identify as a likely error location the mid-circuit data-qubit readout of qubit D2, which results in a phase rotation of qubit D1, see Section IV. Using correlation analysis for simulated data, we identify that $\hat{Y}_{\mathrm{L1}}\hat{X}_{\mathrm{L2}}$ is flipped by the same edges that also flip $\hat{S}^{\mathrm{Z1}}$. The observable $\hat{X}_{\mathrm{L1}}\hat{Y}_{\mathrm{L2}}$ is flipped by the edges that flip $\hat{S}^{\mathrm{Z1}}$ or $Z_{\mathrm{L1}}Z_{\mathrm{L2}}$, but not both.

Using this decoding strategy, we obtain a larger absolute value of these two observables: $\hat{X}_{\mathrm{L1}}\hat{Y}_{\mathrm{L2}}$ takes the value of -0.079 compared to -0.056 for no decoding, and $\hat{Y}_{\mathrm{L1}}\hat{X}_{\mathrm{L2}}$ takes the value of -0.089 compared to -0.023 for no decoding. The larger decoded absolute values of these observables compared to the raw values are consistent with the discussed error mechanism.

For input states with non-zero expectation values of the X_{L} or Y_{L} observables, the split operation yields non-zero expectation values for the $X_{\mathrm{L1}}I_{\mathrm{L2}}$, $I_{\mathrm{L1}}X_{\mathrm{L2}}$, $Y_{\mathrm{L1}}Z_{\mathrm{L2}}$, and $Z_{\mathrm{L1}}Y_{\mathrm{L2}}$ observables, which are unambiguously flipped by errors corresponding to purple edges shown in Fig. S3. These edges are associated with phase-flip errors occurring during the operation of the distance-three surface code and can be corrected.

For the arbitrary state preparation scheme, described in detail in Section VII, the first round of weight-four syndromes is non-deterministic, and as a result unavailable, while all the weight-two syndromes are deterministic (including σ_0^{X1} and σ_0^{X4}). This results in a loss of fault-tolerance due to ambiguous boundary edges and undetectable errors during initialization. The appearance of these ambiguous boundary edges and undetectable errors arises from the loss of the deterministic weight-four stabilizers (σ_0^{Z2} and σ_0^{Z3}) in the first round. In the current work, we do not attempt to modify the syndrome graph, but instead postselect on runs where no non-trivial syndromes are detected in the initial cycle.

Summarizing, in this work, we separately correct phase-flip and bit-flip errors using the X and Z syndrome graphs, respectively, wherever possible. For the arbitrary state preparation, we do not correct errors but discard datasets where non-trivial syndromes are triggered during the preparation cycle.

IV. COHERENT ROTATION QUANTIFICATION AND CORRECTION

In this section, we discuss the single-logical-qubit phase rotation observed in the logical state tomography (Fig. 2a) and logical process tomography (Fig. 4). Applying a 0.11π rotation around the Z axis on the first logical qubit improves the fidelity of the Bell state from 0.780(4) to 0.788(4), and the fidelity of the process from 0.781(11) to 0.812(11), after postselection for no detected errors. In Fig. S4 we present the Pauli transfer matrix with and without the phase rotation correction. The unintended phase rotation has the strongest effect on the highlighted matrix elements. For an ideal split operation without phase errors, the entries highlighted in purple should be zero. The effect of the rotation is that these entries increase in magnitude, while entries with the same input operator outlined in orange decrease. After applying the correction, the absolute values of the unintended

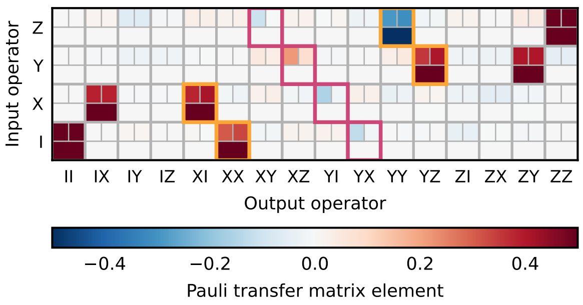

FIG. S4: Postselected Pauli transfer matrix (PTM) for the split operation with and without correction of the phase error on the first logical qubit. Each element in the PTM is color-coded. The bottom part of each cell shows the PTM entry corresponding to an ideal split operation, the left upper part of the cell corresponds to the corrected entry, and the right upper part of the cell corresponds to the uncorrected entry. The entries most affected by the rotation are indicated by colored wireframes, orange for entries whose magnitude is reduced due to the rotation, and purple for entries whose magnitude is increased due to the rotation.

entries outlined in purple are reduced. The deviations of the remaining entries in the PTM from the expected value of zero are within a 95% confidence interval. After postselection for no detected errors, only 142 out of 3 062 runs per prepared state and measurement basis remain.

For the logical Bell state, the final state of the data qubits is, up to a Pauli-frame update, a GHZ state: $(|0\rangle^{\otimes 6}+|1\rangle^{\otimes 6})/\sqrt{2}$. The phase of the non-zero off-diagonal matrix element of the measured state is the sum of the individual single-qubit phase rotations of the data qubits. Therefore, a phase shift on any of the data qubits will result in a phase shift of the logical state.

We consider the most likely error location to be the mid-circuit data-qubit readout operation. The error mechanism is a readout-induced AC-Stark shift of the qubit D1 due to the readout of data qubit D2. The readout resonators of qubits D1 and D2 share the same feedline, and have close readout frequencies. Readout-induced AC-Stark shifts and dephasing have previously been observed in frequency-multiplexed readout schemes [11].

To support our hypothesis that the location of the coherent error is the mid-circuit data qubit readout, we perform reverse-propagation of the phase error from the readout. Before the tomographic readout of the data qubits, the phase error is described by a unitary operator $e^{i\phi\hat{Z}/2}$. During the bit-flip repetition-code stabilizer measurements, the data qubits undergo only CZ gates and echo pulses. The effect of echo pulses is that they revert the sign of the phase error. The unitary operator of the CZ gates commutes with the unitary operator of the phase error. The latest position in the gate sequence where the phase error propagates to another qubit is the CZ gate inside the final X-type stabilizer measurement circuit. At this position, the rotation around the Z axis is transformed into a rotation around the X axis by the $\pi/2$ pulse. In this form, the error no longer commutes with CZ gates. After the CZ gate between D1 and X2, the error unitary is no longer confined to a single qubit. Further backpropagation leads into a measurement operation for X2, at which point the error should be detected. The locations in the gate sequence, together with the frame-updated errors at those locations, are shown in Fig. S5.

We note that due to the echo pulses during the Z-type stabilizer measurements in the bit-flip repetition code part of the circuit, any systematic error that repeats in the same way during the single-qubit or two-qubit gates during the Z-type stabilizer measurements would cancel out. The locations in the circuit containing these operations are shown in light green.

V. SIMULATION

To estimate logical error rates for the gate sequence used in the experiment (Fig. 5), we use a Monte-Carlo simulation of the 17-qubit wavefunction. Our noise model incorporates single- and two-qubit depolarizing channels for the corresponding gates. The depolarization probabilities are determined from error rates measured using randomized benchmarking for single-qubit gates and interleaved randomized benchmarking for two-qubit gates (see Methods), and are performed individually for each qubit and qubit pair. Additionally, we include a coherent phase rotation on qubit D1 during the mid-circuit data qubit readout, as discussed in Section IV. However, we do not include the effect of leakage in our simulations, as in our experiments such events are postselected and the remaining data are assumed to be described by a noise model without leakage (see Section II for details on leakage in the context of the state preservation experiment). For each readout basis, we sample 5×10^4 experimental outcomes.

Readout errors are simulated by applying a Pauli-X gate before a perfect measurement, with the probability determined by the experimentally characterized two-state readout assignment error. Initialization errors are modeled as an imperfect readout operation applied to an ideal ground state. At each step of the circuit, if a qubit is not involved in an active operation, a Pauli error channel based on the coherence properties of the qubit is applied. The probabilities for this error channel are given by $p_i = 1 - e^{-t/T_i}$, with $T_X = T_Y = 4T_1$, and $T_Z = T_\varphi$. The energy relaxation time T_1 and Hahn echo time $T_{2,E}$ are experimentally determined for each qubit, and the

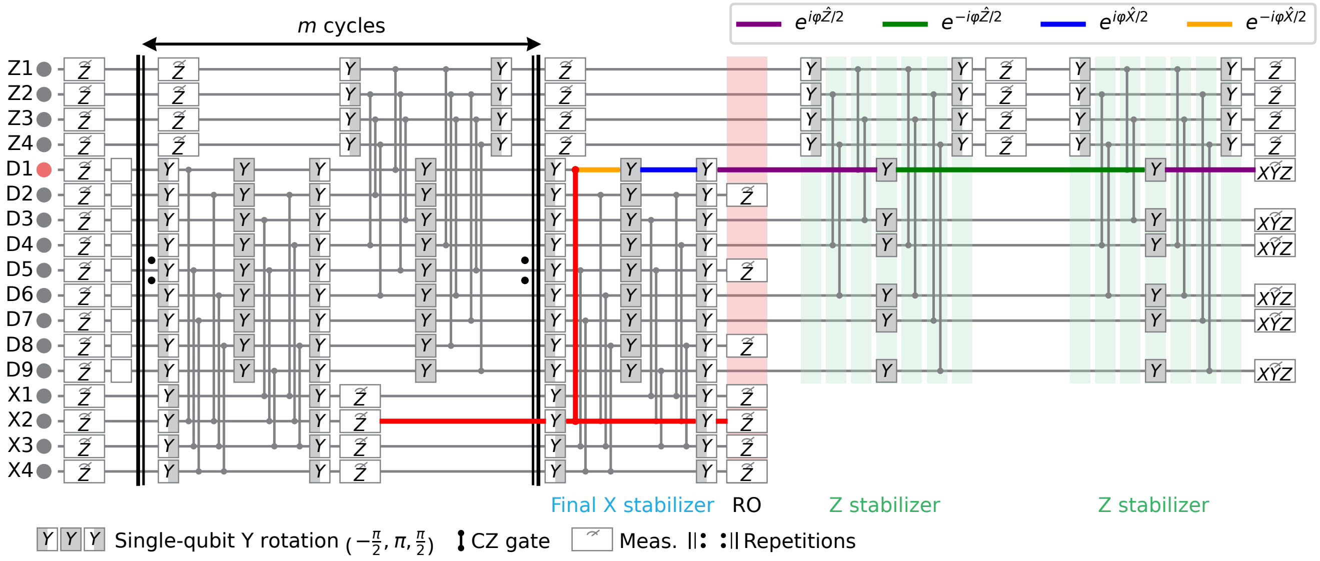

FIG. S5: Gate sequence of the logical Bell state preparation experiment. The circuit locations where a single-qubit coherent error can result in the observed logical qubit phase rotation are indicted by purple, green, blue and orange solid lines. The colors indicate the frame-updated error unitary corresponding to a specific location. The CZ gate which propagates the error onto the X2 auxiliary qubit, together with the measurements whose parity is affected by such an error is shown in red. The light-red background indicates the mid-circuit data qubit readout.

pure dephasing rate is computed as

$$\frac{1}{T_\varphi} = \frac{1}{T_{2,E}} - \frac{1}{2T_1}.$$

The value of t corresponds to the gate duration as used in the experimental implementation. We use Ramsey echo time, rather than the decoherence time T_2^*, as our experimental implementation incorporates echo pulses that cancel the contribution of slow frequency drifts. Other known noise sources, such as drive crosstalk, flux crosstalk, measurement-induced dephasing are not included in the noise modeling and simulations.

As a consistency check, we evaluate the simulated average syndrome values for bulk error correction cycles in the surface code memory experiment using the error model based on individually measured single-qubit and two-qubit error rates. For weight-two stabilizers, the simulated average is 0.146, and for weight-four stabilizers, it is 0.230. These values are higher than those observed experimentally (see Section II). We attribute this discrepancy in part to the fact that our two-qubit randomized benchmarking tends to overestimate gate errors compared to those occurring during actual surface code cycles. This is because benchmarking is performed for each qubit pair individually, while two-qubit gates are tuned in groups, as executed in the surface code memory experiment. Additionally, slow drifts in gate performance and coherence times – e.g., due to two-level system defects – may also contribute to the observed mismatch.

We quantify the agreement between experimental and simulated data shown in Fig. 2a by computing the norm-based fidelity [12] between the simulated and experimentally extracted logical density matrices as

$$\mathcal{F}_{\mathrm{overlap}} = \mathrm{Tr}[\hat{\rho}_{\mathrm{sim}}\hat{\rho}_{\mathrm{exp}}]/\max\left[\mathrm{Tr}\,\hat{\rho}_{\mathrm{sim}}^2, \mathrm{Tr}\,\hat{\rho}_{\mathrm{exp}}^2\right]$$

and find $\mathcal{F}_{\mathrm{overlap}} = 0.972(3)$ for raw data, $\mathcal{F}_{\mathrm{overlap}} = 0.956(3)$ for decoded data, and $\mathcal{F}_{\mathrm{overlap}} = 0.954(10)$ for postselected data.

To gain insights into the effect of reductions in error rates, we scale all Pauli error probabilities by a constant factor of $1/x$. For the remainder of this section, we omit the coherent error, resulting in a circuit composed solely of Clifford gates and Pauli errors. Simulations are performed using the Python package stim [13]. For each improvement factor and logical observable, we sample 10^7 experiment outcomes, from which we calculate the corresponding logical operator expectation values. For all three observables, the raw logical observable error rates scale inversely with the improvement factor (see Fig. S6a, b, and c). By discarding simulated outcomes where one or more syndrome elements are non-zero, we obtain postselected observable values. For the $Z_{\mathrm{L}1}Z_{\mathrm{L}2}$ observable, we find an inverse cubic scaling of the postselected error rate with the improvement factor compared to the inverse linear scaling of the raw error rate. This is consistent with the expected behavior of a protocol where the smallest undetectable error weight causing a logical error is three. For the other observables, while postselection improves the error rates, the scaling remains inverse linear.

The matching graph weights for simulation-based decoding are derived from the probabilities within the detector error model of a stim circuit with the specified noise

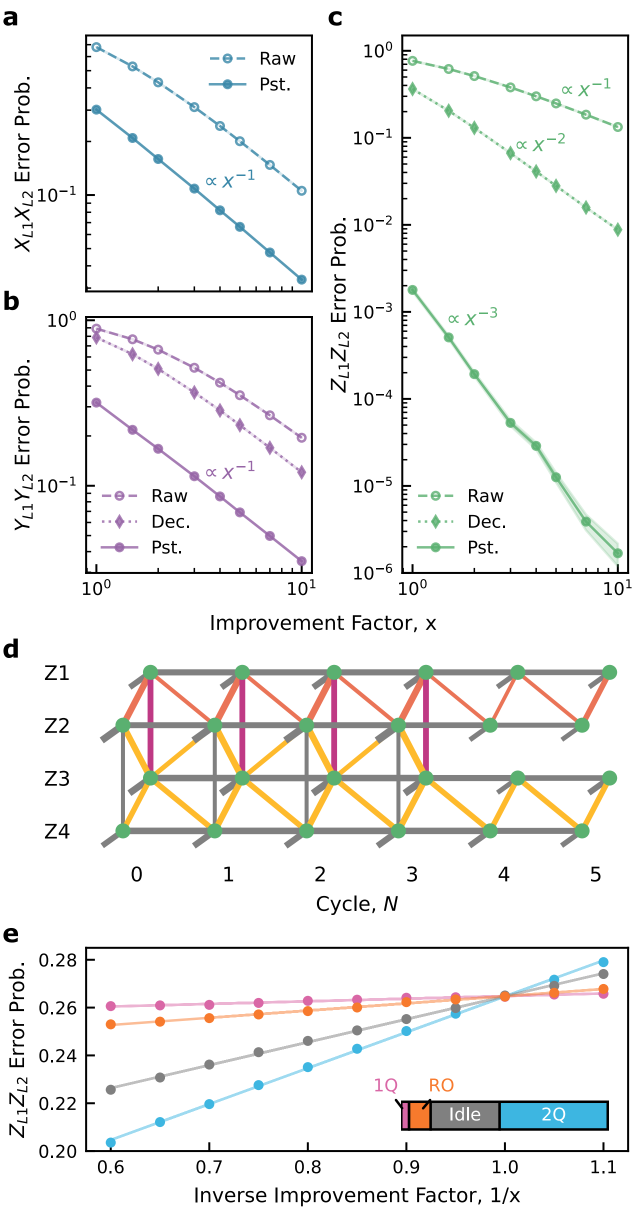

FIG. S6: Results of the stabilizer simulation of the lattice-split protocol. **a, b, c** Raw, decoded and postselected expectation values of (**a**) $X_{\mathrm{L}1}X_{\mathrm{L}2}$, (**b**) $Y_{\mathrm{L}1}Y_{\mathrm{L}2}$, and (**c**) $Z_{\mathrm{L}1}Z_{\mathrm{L}2}$ as a function of error rate improvement factor that scales down uniformly all modeled physical noise sources. The shaded regions indicate the 68% confidence intervals and markers the means obtained from Monte Carlo sampling. **d** Matching graph derived from the error model, with circles corresponding to syndrome elements and edges to possible errors, with their probability encoded in the thickness of the edge. Color coding as in Fig. 2b. **e** Scaling of the logical $Z_{\mathrm{L}1}Z_{\mathrm{L}2}$ error rate when improving single-qubit (pink), readout (orange), idling (gray) and two-qubit errors (cyan), with relative contributions to the logical error rate of 3.8%, 10.5%, 33.4% and 52.4% respectively.

model. The resulting graph is qualitatively similar to the experimental one shown in Figure 2b. We note that for the Z-type syndrome graph we do not need to include the coherent phase error on qubit D1, since this error does not affect Z syndromes.

Comparing the connectivity of both graphs, we recover the absence of significant correlations between the syndromes of the two repetition codes after the split operation, as can be seen by the missing edges connecting the stabilizer qubits Z1, Z2 with Z3, Z4 for cycles $N = 4, 5$, see Fig. S6d. A key difference is the absence of the weight-two time-like correlations, which are, on the other hand, present in the experimental decoding graph. In our gate sequence, which does not feature auxiliary-qubit reset after readout, such correlations can be caused by readout misclassification errors or data-qubit leakage [7]. Both of these error mechanisms are absent in our simulation. As with the experimental data, we apply minimum-weight perfect matching decoding using the matching graph. This yields an inverse quadratic error scaling for the decoded $Z_{\mathrm{L}1}Z_{\mathrm{L}2}$ observable, as expected for a fault-tolerant operation. We also observe some improvement in the decoded error rate of the $Y_{\mathrm{L}1}Y_{\mathrm{L}2}$ observable, compared to the raw error rate, due to the correction of bit-flip errors during the protocol.

To identify the contribution of different error mechanisms, we simulate the dependence of the decoded logical $Z_{\mathrm{L}1}Z_{\mathrm{L}2}$ error rate on the individual error mechanisms: single-qubit gate errors, two-qubit gate errors, idling errors and readout errors. Following the error contribution analysis approach introduced in Ref. [14], we fit a linear slope to each simulation sweeping one noise parameter at a time. We then linearize the obtained dependence at the point where the noise is equal to our device model, i.e., $x = 1$. From the extracted slopes, we break down the marginal error of the $\hat{Z}_{\mathrm{L}1}\hat{Z}_{\mathrm{L}2}$ observable into individual error contributions. We conclude that the most significant error mechanisms are two-qubit-gate and idling errors. The breakdown and the scaling of the error on the individual error mechanisms are shown in Fig. S6e.

We also simulate the Pauli transfer matrix (PTM) reconstruction experiment using the same gate error model as above without the coherent rotation on D1, and with an initial state preparation circuit adapted to match the arbitrary state preparation protocol. Following the experimental post-processing procedure, runs with errors detected during the first cycle of stabilizer measurements are discarded. The PTM reconstructed from the simulated data is shown in Fig. S7. We observe qualitative agreement with the experimentally extracted PTM with the compensated phase error (see Fig. 4). The overlap fidelities between the experimental process maps and the simulated ones are $\mathcal{F}_{\text{overlap}} = 0.955(6)$ for the raw data, $\mathcal{F}_{\text{overlap}} = 0.956(6)$ for the decoded data, and $\mathcal{F}_{\text{overlap}} = 0.984(12)$ for the postselected data, indicating good agreement between experimental data and simulations. This suggests that the employed multi-parameter noise model captures the dominant error mechanisms that

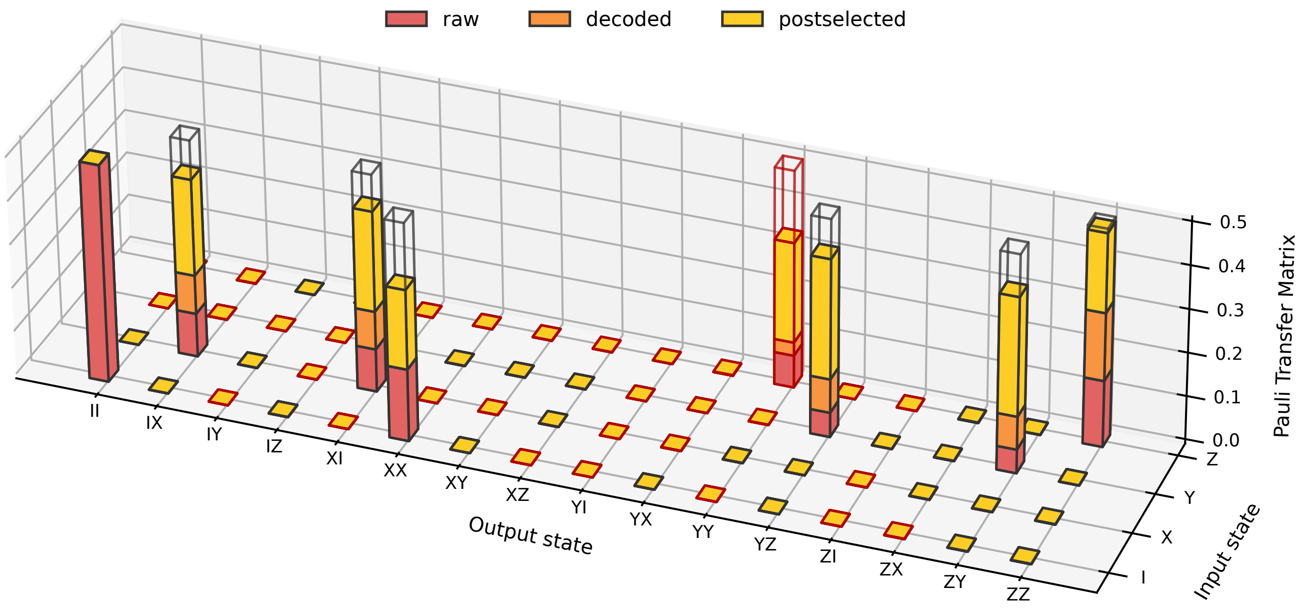

FIG. S7: Simulated Pauli transfer matrix of the split operation reconstructed from simulated raw, decoded and postselected logical observable outcomes. The designations match those of Fig. 4.

currently limit the performance of our setup.

VI. DISTANCE-ONE IMPLEMENTATION OF BELL-STATE PREPARATION PROTOCOL

To compare the bit-flip error-protected protocol with a non-encoded circuit, we implement a distance-one split protocol. In this process, we generate a Bell state between two physical qubits within the three-by-three lattice of data qubits. This entanglement is created by performing X-type stabilizer measurements with a third, central data qubit. We select the subset of data and auxiliary qubits exhibiting the best performance. In this encoded protocol, all three data qubits, D7, D8, and D9, are initialized in the $|0\rangle$ state, followed by X-stabilizer measurements on qubits X3 and X4 to distribute the entanglement, as illustrated in Fig. S8a. After $1 \leq m+1 \leq 6$ cycles of syndrome extraction, we read out the central data qubit D8 in the Z basis (see Fig. S8b). For the remaining two physical qubits, we perform a tomographic readout (see Fig. S8c), updating the Pauli frame based on the readout of qubit D8 and the two X-stabilizer measurement outcomes. The quantum circuit for this protocol is shown in Fig. S8d.

When plotting the leakage-rejected and Pauli-frame-updated expectation value of $X_{\mathrm{L1}}X_{\mathrm{L2}}$ as a function of the number of X-syndrome extraction cycles m, we observe a value close to 0.7, independent of the number of cycles, as shown by the empty circles in Fig. S8e. This is because, for the Pauli-frame update of $X_{\mathrm{L1}}X_{\mathrm{L2}}$ only the most recent X-stabilizer values are used, and not those from earlier cycles. By postselecting runs with consistent X-stabilizer values across all cycles, we operate this distance-one implementation in a phase-flip error detection mode. For the postselected data, we find that, when only one round of stabilizer extraction is performed, postselection does not enhance the observable value. This is because the X-stabilizer values for qubits initialized in $|0\rangle$ are not predefined and become fixed only after the first round of stabilizer measurements. As a result, the initial round cannot be used for postselection. However, in subsequent cycles, we observe improvements in the $X_{\mathrm{L1}}X_{\mathrm{L2}}$ observable value by postselecting on consistent X-stabilizer measurements.

Similarly, we track the $Z_{\mathrm{L1}}Z_{\mathrm{L2}}$ observable as a function of the number of X-stabilizer measurement cycles, m. In this case, we observe a reduction in the raw observable value with m, primarily due to energy relaxation of the data qubits (empty circles in Fig. S8f). Since this distance-one implementation lacks Z-stabilizer measurements, we are neither able to correct nor detect bit-flip errors. For $m+1=4$, corresponding to the same number of cycles used in the distance-three implementation, we find an observable value of 0.591(8) for $Z_{\mathrm{L1}}Z_{\mathrm{L2}}$, indicated by an open star in Fig. S8f. We also observe a modest improvement on the $Z_{\mathrm{L1}}Z_{\mathrm{L2}}$ observable when postselecting on consistent X-stabilizer measurements, which we attribute to Y errors. Y errors can be detected by X syndromes, but affect both X and Z observables.

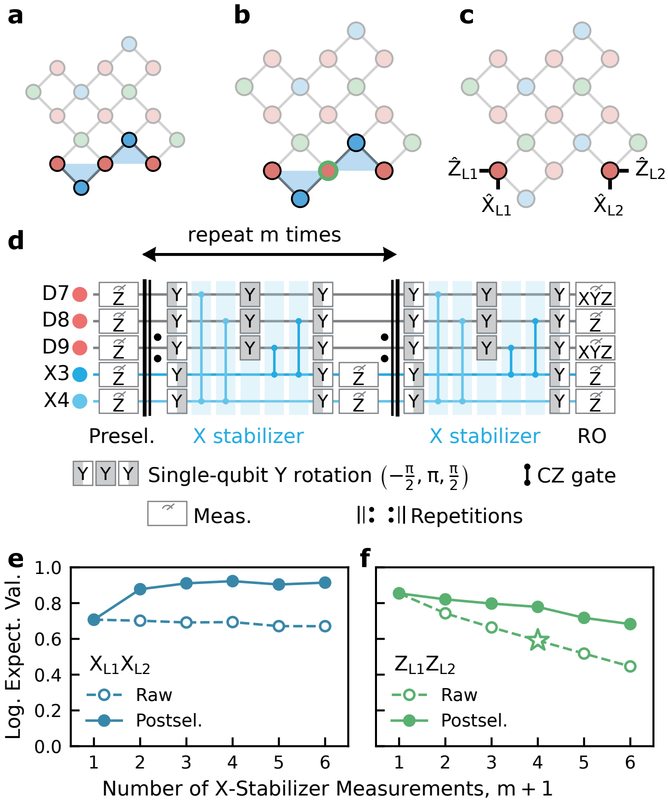

FIG. S8: Distance-one implementation of the lattice-split protocol. **a, b, c** Schematic layout of the qubits used in the distance-one protocol. (**a**) Data qubits D7, D8, D9, used for the Bell-state creation, along with the X-stabilizers X3, X4 mediating the entanglement between D7 and D9, are highlighted. (**b**) Mid-circuit readout of the data qubit D8 indicated as a green circle outline as well as (**c**) logical operator definitions for the two data qubits sharing entanglement after the operation. **d** Quantum circuit for the distance-1 protocol. Raw and postselected expectation values of **e** $X_{L1}X_{L2}$ and **f** $Z_{L1}Z_{L2}$. The star ($\star$) marks the data point corresponding to the same number of repetitions as used in the distance-three implementation in Fig. 2a.

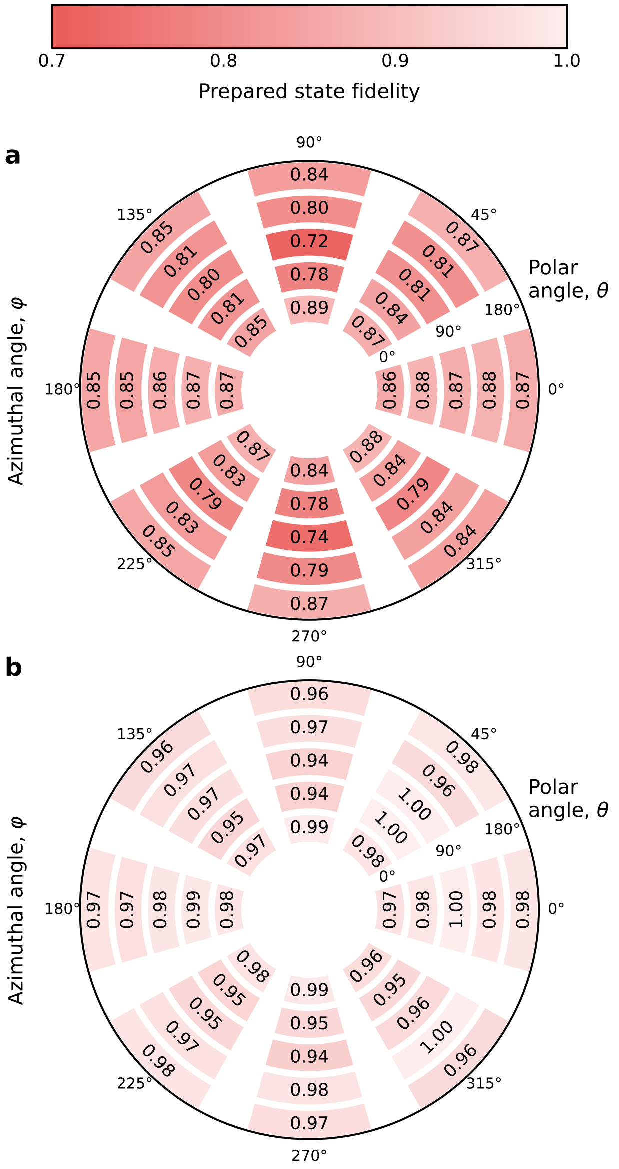

FIG. S9: Arbitrary state preparation and readout performance figures **a** with postselection only on leakage events and **b** with postselection both on leakage and non-trivial syndromes.

VII. ARBITRARY STATE PREPARATION AND TOMOGRAPHY OF A LOGICAL QUBIT

The surface code enables fault-tolerant preparation of the logical $\hat{X}_L$ and $\hat{Z}_L$ eigenstates, as described in Section II. This is possible because errors affecting these states can be corrected using either only X-type or only Z-type syndrome data, respectively. For arbitrary logical states, fault-tolerant preparation requires distillation methods [15]. Nevertheless, it is possible to prepare an arbitrary state of the distance-three surface code non-fault-tolerantly. Here, we follow the approach introduced in [16–18], which yields an incomplete subset of the stabilizers after the preparation. The arbitrary state is injected into the central data qubit (D5). The other data qubits are initialized as illustrated in Fig. 3a, ensuring that all weight-two stabilizers are predefined. This approach allows us to compute four out of eight syndrome elements for the initial cycle of error correction. These syndrome elements do not account for all possible single-qubit and two-qubit gate errors, such as those affecting D5. Moreover, these syndromes are insufficient to determine the effect the errors will have on the logical observables, rendering correction impossible. Nevertheless, by postselecting runs where the first cycle of stabilizer measurements yields the expected outcomes for the weight-two stabi-

lizers, we are able to detect some of the possible errors during state preparation. From a code deformation perspective this preparation can be understood as adding eight data qubits and stabilizers to a surface-code patch consisting only of D5, which encodes the original logical qubit.

Similarly, by applying code deformation to the distance-three surface-code qubit in reverse, we can perform a logical-qubit measurement in an arbitrary basis. All data qubits, except for D5, are read out in the basis of the weight-two stabilizer they are included in. D5 can be read out in any basis. In our experiment, we use logical quantum state tomography, and the arbitrary-basis measurement is only needed for the $\hat{Y}_{\mathrm{L}}$ operator, since $\hat{X}_{\mathrm{L}}$ and $\hat{Z}_{\mathrm{L}}$ are measured in a fault-tolerant manner. The $\hat{Y}_{\mathrm{L}}$ operator is defined as the product of $\hat{Z}_{\mathrm{L}}$ and $\hat{X}_{\mathrm{L}}$, which in terms of data qubits is $\hat{X}_2\hat{Z}_4\hat{Y}_5\hat{Z}_6\hat{X}_8$. To improve the readout fidelity, we discard runs where one of the stabilizers Z1, Z4, X1, or X4 extracted from the final data-qubit readout does not match the final round of stabilizer measurements.

By combining these two methods, we can assess our ability to herald arbitrary logical states and characterize their fidelity using logical quantum state tomography. To achieve this, we prepare the central data qubit (D5) in a desired arbitrary state $|\psi\rangle$, parameterized by the polar and azimuthal angles introduced in Fig. 3b, with the remaining data qubits initialized as described above. We then perform a complete round of Z- and X-type stabilizer measurements to initialize the distance-three surface-code qubit in the arbitrary state. Finally, by measuring the data qubits in the three logical bases $\hat{X}_{\mathrm{L}}$, $\hat{Y}_{\mathrm{L}}$, and $\hat{Z}_{\mathrm{L}}$, as described earlier, we compute the fidelity according to

$$\mathcal{F}_{\psi} = \frac{1}{2}\Big(1 + \langle \hat{X}_{\mathrm{L}}\rangle\langle\hat{\sigma}_x\rangle_{\psi} + \langle \hat{Y}_{\mathrm{L}}\rangle\langle\hat{\sigma}_y\rangle_{\psi} + \langle \hat{Z}_{\mathrm{L}}\rangle\langle\hat{\sigma}_z\rangle_{\psi}\Big), \tag{S6}$$

where $0 \leq \langle \hat{X}_{\mathrm{L}}\rangle, \langle \hat{Y}_{\mathrm{L}}\rangle, \langle \hat{Z}_{\mathrm{L}}\rangle \leq 1$ represent the logical readout results in the respective basis. Fidelities exceeding unity are clipped to ensure positive semi-definiteness of the density matrix. The fidelities obtained using this method are shown in Fig. S9, both for the case of leakage rejection only (a) and the case of leakage rejection combined with postselection on no detected syndrome events (b). Without syndrome postselection, we achieve an average fidelity of 83.5%, which improves to 97.0% when error detection is applied. We observe that the lowest fidelities occur for the $\hat{Y}_{\mathrm{L}}$ eigenstates, specifically for $\theta = 90°$ and $\varphi = \pm 90°$, where both phase-flip and bit-flip errors contribute to the infidelity. Averaging over all angles, out of the initial 3 322 repetitions, 3 044 remain after leakage rejection, and about 1 577 remain after postselection on no syndrome events.

[1] Horsman, C., Fowler, A. G., Devitt, S., and Meter, R. V., Surface code quantum computing by lattice surgery, New Journal of Physics **14**, 123011 (2012).

[2] Krinner, S., Lacroix, N., Remm, A., *et al.*, Realizing repeated quantum error correction in a distance-three surface code, Nature **605**, 669 (2022).

[3] Hesner, I., Hetényi, B., and Wootton, J. R., Using detector likelihood for benchmarking quantum error correction, arXiv:2408.02082 (2024).

[4] Google Quantum AI, Suppressing quantum errors by scaling a surface code logical qubit, Nature **614**, 676 (2023).

[5] Acharya, R., Abanin, D. A., Aghababaie-Beni, L., *et al.*, Quantum error correction below the surface code threshold, Nature 10.1038/s41586-024-08449-y (2024).

[6] Ryan-Anderson, C., Bohnet, J. G., Lee, K., *et al.*, Realization of real-time fault-tolerant quantum error correction, Phys. Rev. X **11**, 041058 (2021).

[7] Remm, A., Lacroix, N., Bödeker, L., *et al.*, Experimentally informed decoding of stabilizer codes based on syndrome correlations, arXiv:2502.17722 (2025).

[8] Spitz, S. T., Tarasinski, B., Beenakker, C. W. J., and O'Brien, T. E., Adaptive weight estimator for quantum error correction in a time-dependent environment, Advanced Quantum Technologies **1**, 1800012 (2018).

[9] Edwards, S. F. and Anderson, P. W., Theory of spin glasses, Journal of Physics F: Metal Physics **5**, 965 (1975).

[10] Higgott, O., PyMatching: a Python package for decoding quantum codes with minimum-weight perfect matching, ACM Transactions on Quantum Computing **3**, 16 (2022).

[11] Heinsoo, J., Andersen, C. K., Remm, A., *et al.*, Rapid high-fidelity multiplexed readout of superconducting qubits, Phys. Rev. Appl. **10**, 034040 (2018).

[12] Liang, Y.-C., Yeh, Y.-H., Mendonça, P. E. M. F., *et al.*, Quantum fidelity measures for mixed states, Reports on Progress in Physics **82**, 076001 (2019).

[13] Gidney, C., Stim: a fast stabilizer circuit simulator, Quantum **5**, 497 (2021).

[14] Chen, Z., Satzinger, K. J., Atalaya, J., *et al.*, Exponential suppression of bit or phase errors with cyclic error correction, Nature **595**, 383 (2021).

[15] Bravyi, S. and Kitaev, A., Universal quantum computation with ideal Clifford gates and noisy ancillas, Phys. Rev. A **71**, 022316 (2005).

[16] Ye, Y., He, T., Huang, H.-L., *et al.*, Logical magic state preparation with fidelity beyond the distillation threshold on a superconducting quantum processor, Phys. Rev. Lett. **131**, 210603 (2023).

[17] Li, Y., A magic state's fidelity can be superior to the operations that created it, New Journal of Physics **17**, 023037 (2015).

[18] Lao, L. and Criger, B., Magic state injection on the rotated surface code, *Proceedings of the 19th ACM International Conference on Computing Frontiers*, CF 22, 113–120 (2022).
